# Clinical-like cryotherapy in acute knee arthritis of the knee improves inflammation signs, pain, joint swelling, and motor performance in mice

**DOI:** 10.1371/journal.pone.0261667

**Published:** 2022-01-21

**Authors:** Paula A. T. S. Castro, Germanna M. Barbosa, Dafiner H. Machanocker, Raphael S. Peres, Thiago M. Cunha, Jonathan E. Cunha, Francisco F. B. Oliveira, Fernando Silva Ramalho, Thiago L. Russo, Fernando Q. Cunha, Tania F. Salvini

**Affiliations:** 1 Department of Physical Therapy, Center of Biological Sciences and Health (CBSH), Federal University of São Carlos (UFSCAR), São Carlos, Brazil; 2 Department of Pharmacology, Center for Research in Inflammatory Diseases (CRID), Ribeirão Preto Medical School, University of São Paulo (USP), Ribeirão Preto, Brazil; Universidade Federal de Pernambuco, BRAZIL

## Abstract

To assess the effects of clinical-like cryotherapy on inflammatory signs (*in vivo* neutrophil migration, cytokines, and joint inflammation), pain, joint swelling, balance, and motor coordination in mice with knee arthritis. Young C57BL/6 mice were randomly divided into three groups (8 to 10 mice per group): Control group: mice with no intervention; antigen-induced arthritis (AIA) group: mice sensitized and immunized with intra-articular (i.a.) injection of methylated bovine serum albumin (mBSA); and AIA + cryotherapy group: mice sensitized, immunized with i.a. injection of mBSA, and submitted to a clinical-like cryotherapy protocol. After 21 days of sensitization, AIA and AIA + cryotherapy groups received i.a. injection of mBSA (100 μg/joint) to induce joint inflammation, and a clinical-like cryotherapy protocol was applied to AIA + cryotherapy group (crushed ice bag, two cryotherapy sessions of 20 min every two hours). Experimental analysis was conducted in the initial (immediately after i.a. injection of mBSA) and final periods (two hours after the second cryotherapy session). The number of synovial fluid neutrophils, cytokine levels, joint histology, pain, joint swelling, and motor performance were also analyzed. Our results showed that clinical-like cryotherapy in mice with acute knee arthritis reduced inflammatory signs, pain, and joint swelling, and improved balance and motor coordination.

## Introduction

Arthritis is characterized by infiltration of inflammatory cells and cartilage and destruction of bone, clinically manifested as pain, swelling, and stiffness in affected joints [1]. Inflammatory cytokines and chemokines play a pivotal role in local and systemic inflammation in patients with arthritis, contributing to development and progression of the disease [2]. Although not well studied, neutrophils also participate in disease progression, and evidence indicates that neutrophil influx occurs during disease recurrence [3]. The experimental model of antigen-induced arthritis (AIA) is suitable, reproducible, and exhibits histopathological findings similar to human rheumatoid arthritis [4,12].

Several drug classes are used to treat arthritis, relieve symptoms, and avoid disease progression [5]. Although some components of the arthritic inflammatory response still need to be elucidated, significant developments have been observed in recent decades, including noninvasive therapeutic agents targeting inflammation. Cryotherapy is often used as non-pharmacological intervention to decrease pain, swelling, and inflammation [6]; its physiological effects decrease local blood flow by vasoconstriction, tissue metabolism, oxygen use, and muscle injury [7]. Therefore, studying the possible effects of cryotherapy in an arthritis model may be useful for clinical practice. For example, the ice pack was considered well-tolerated [8,37] and effective in reducing joint and muscle damage in chronic models of anterior cruciate ligament transection [9].

Thus, this study aimed to assess the effects of clinical-like cryotherapy on inflammatory signs (i.e., *in vivo* neutrophil migration, cytokines, and joint inflammation), pain, joint swelling, balance, and motor performance in mice with knee arthritis. We hypothesized that clinical-like cryotherapy reduces inflammatory signs, pain, and joint swelling and improves balance and motor coordination in mice with acute knee arthritis.

## Materials and methods

This study was conducted following the Guide for the Care and Use of Laboratory Animals from the National Institutes of Health and the International Association for the Study of Pain. The protocol was approved by the research ethics committee of University of São Paulo (Protocol Number: 9197020816) and Federal University of São Carlos (Protocol Number: 1124010316/2015). Trained professionals blinded to experimental groups conducted all procedures. The study was designed to detect a between-group difference of one second in paw withdrawal latency based on a previous study with similar methods [10]. A statistical power (1-β) of 80%, alpha of 5%, and effect size of 0.52 were considered for sample calculation, resulting in a total of 30 mice (8 to 10 per group). The G*Power software (version 3.1; University of Trier, Trier, Germany) was used for sample size calculation and power analysis [11].

### Animals and experimental design

The study was performed using 30 young male C57BL/6 mice (20 to 25 g), maintained in temperature-controlled rooms (22 to 25°C), and provided with water and food *ad libitum*. Mice were randomly distributed into three experimental groups (8 to 10 mice each): control group, mice with no intervention; AIA group, mice were sensitized and immunized with i.a. injection of mBSA; AIA + Cryotherapy group, mice were sensitized, immunized with i.a. injection of mBSA, and submitted to the clinical-like cryotherapy protocol. After intra-articular (i.a.) injection of methylated bovine serum albumin (mBSA, 100 μg/joint) to induce joint inflammation, this study was divided into two periods of experimental analysis: initial, immediately after i.a. injection of mBSA; and final, two hours after the second cryotherapy session.

### Induction of antigen-induced arthritis

AIA and AIA+Cryotherapy mice were immunized as previously described [12–14] and sensitized with 500 μg of mBSA in 0.2 mL of an emulsion containing 0.1 mL of saline and 0.1 mL of Freund’s Complete Adjuvant (1 mg/mL of inactive *Mycobacterium tuberculosis*). The application was administered by subcutaneous injection in the dorsal region (near the tail) on day 0. Mice were boosted with the same preparation on days 7 and 14. Control mice were neither sensitized nor immunized with i.a. injection of mBSA. Twenty-one days after initial injection, arthritis was induced in immunized mice with i.a. injection of mBSA (100 μg/joint), dissolved in 10 μL of saline into the right tibiofemoral joint. Mice were manipulated with extreme caution and anesthetized (O_2_: 2.0 L/m, 2% isoflurane) during one min for each immunization to minimize pain. AIA and AIA+Cryotherapy mice immunized with i.a. injection of mBSA were isolated and placed in a separate cage. Mice from all groups were anesthetized with O_2_ 2.0 L/m, 2% isoflurane and euthanized by cervical dislocation in the final period of experimental analysis.

### Clinical-like cryotherapy protocol

**Fig 1:**
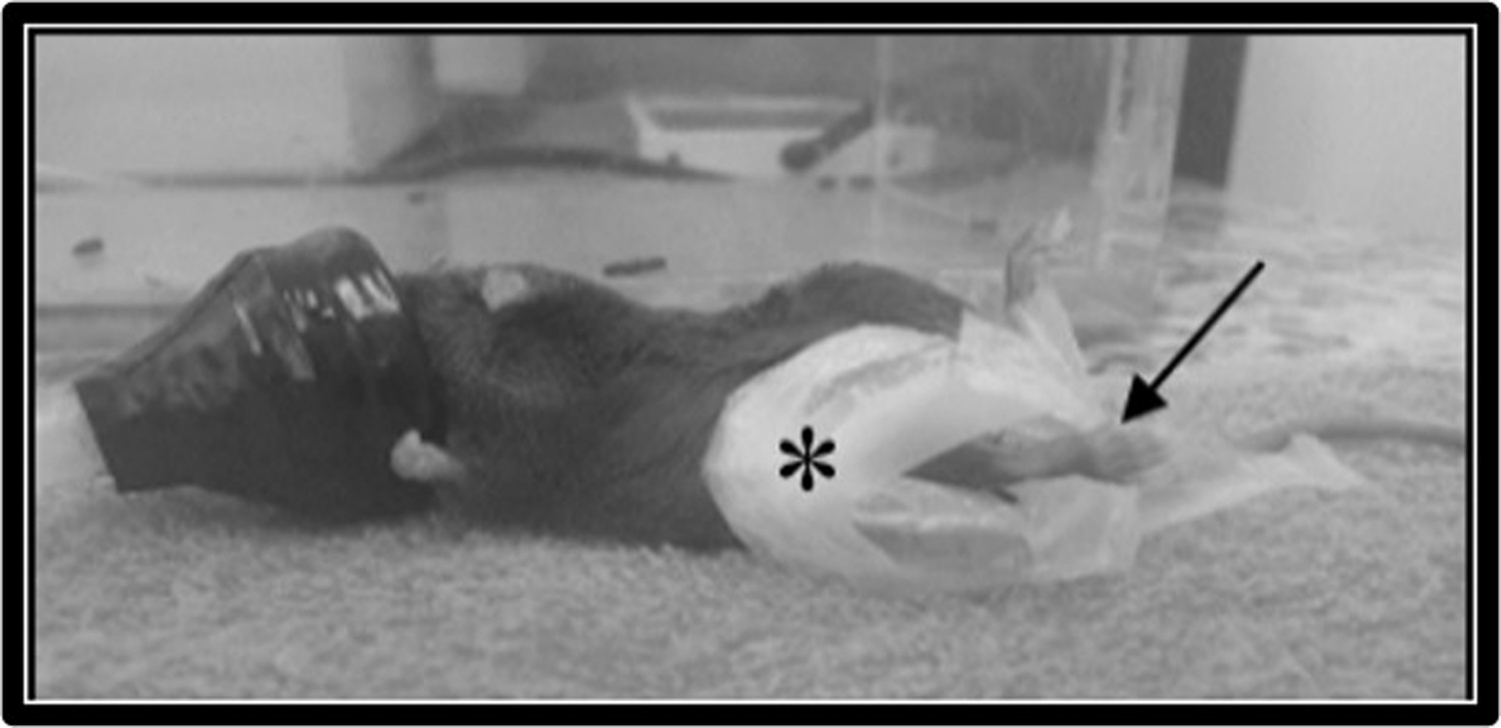
Position of mice during clinical-like cryotherapy. *crushed ice bag; → right paw.

This protocol was adapted to simulate clinical practice [37]. First, mice were anesthetized (O_2_ 2.0 L/m, 2% isoflurane) and maintained in dorsal decubitus on a table with right knee in lateral position (Fig 1). Then, clinical-like cryotherapy (plastic bag filled with crushed ice) was applied directly to the right knee immediately after i.a. injection of mBSA. Two cryotherapy sessions were performed for 20 min every two hours.

### *In vivo* neutrophil migration

In the final period of experimental analysis, the articular cavity of immunized (AIA group, AIA + Cryotherapy group) and non-immunized (control group) mice were analyzed. Neutrophil migration was determined through synovial fluid, using a previously established method (with modifications) [15,16]. Articular cavities were washed twice with 3.3 μL of phosphate-buffered saline (PBS) containing 1 mM ethylenediaminetetraacetic acid (EDTA) and diluted to a final volume of 50 μL with PBS/EDTA to assess leukocyte migration. Neutrophils were counted in a Neubauer chamber with Turk’s solution, and results were expressed as number of neutrophils (mean ± standard error of mean [SEM]) per joint cavity.

### Cytokine levels

Concentrations of IL-1β, IL-10, TNF-α, and IL-6 were measured using commercial enzyme-linked immunosorbent assay (ELISA) kits (DuoSet; R&D Systems, Minneapolis, MN, USA). In the final period of experimental analysis, mice were terminally anesthetized, and tibiofemoral joints and synovial membranes were removed and homogenized in 300 μL of buffer with protease inhibitors, as previously described (with modifications) [17,18]. Results were expressed as pg/ml of each cytokine.

### Evaluation of joint inflammation

Tibiofemoral joints were collected in the final period of experimental analysis, fixed in 4% (vol/vol) buffered formalin, and decalcified in 10% EDTA for two to three weeks. Tissues were then trimmed, dehydrated in ethanol, and embedded in paraffin for histological preparation. Joint sections were stained with hematoxylin and eosin (H&E) to analyze synovitis (i.e., inflammatory cell influx and synovial hyperplasia). Synovitis severity was classified by measuring thickness of synovial cell layer on a scale from 0 to 3 (0  =  1 to 2 cells, 1  =  2 to 4 cells, 2  =  4 to 9 cells, and 3  =  ≥ 10 cells) and cellular density in the synovial stroma on a scale from 0 to 3 (0  =  normal cellularity, 1  =  slightly increased cellularity, 2  =  moderately increased cellularity, and 3  =  greatly increased cellularity) [19]. Three histological sections were assessed separately, obtaining an average value for each mice.

### Dorsal flexion of tibiofemoral joint: Assessment using a modified electronic pressure-meter test for mice

Articular nociception of the tibiofemoral joint was assessed in the final period of experimental analysis using a previously established method (with modifications) [9,20]. Mice were placed in acrylic cages (12 × 10 × 17 cm) with a wire grid floor in a quiet room 15 to 30 min before testing for environmental adaptation. Stimulations were applied when animals were quiet, not exploring, defecating, or resting on their paws. An electronic pressure-meter and hand-held force transducer fitted with polypropylene tip (Insight EFF 301, Brazil) were used in these experiments. For this model, a large tip (4.15 mm^2^) was attached to the probe. An increasing perpendicular force was applied to the central area of plantar surface of the hind paw to induce flexion of tibiofemoral joint, followed by paw withdrawal. A tilted mirror below the grid provided a clear view of the hind paw. The electronic pressure-meter automatically recorded the intensity of force applied when the paw was withdrawn. The test was repeated until three consistent measurements (i.e., variation of less than 1 g) were obtained. Flexion-elicited mechanical threshold was expressed in grams (g).

### Joint swelling (edema)

Joint thickness of the knee was analyzed under anesthesia (O_2_ 2.0 L/m, 2% isoflurane) using an electronic digital caliper (Mitutoyo Absolute Digimatic 150 mm, Japan) and measured in the initial and final periods of experimental analysis in the same conditions. Three measurements were performed in the same area for each joint, and results were expressed in mm [12,21,22].

### Knee skin temperature

Knee skin temperature was analyzed under anesthesia (O_2_ 2.0 L/m, 2% isoflurane) using an infrared digital thermometer (-18 to 260°C; Wurth Temp, Cotia, Brazil) in the initial and final periods of experimental analysis. Three measurements were performed in the same area for each joint, and results were expressed in°C [15,21].

### Gait function—balance and motor coordination

Gait function (balance and motor coordination) was assessed using a previously established method (with modifications) [23,24]. Each mouse was placed in an individual compartment of the rotarod (Insight EFF 412, Brazil) and forced to keep walking to avoid falling off the rotarod. Fall time was recorded in seconds (i.e., latency time). Mice that did not fall off the rotarod received the maximum score of 300 s. Each mouse performed low rotation (8 rpm) for 300 s, 48 hours before the initial period of experimental analysis to adapt and attain stable performance in the rotarod. On the experimental day, low speed (10 rpm) was established, and mice performed the test until 300 s or falling [24]. Analysis was performed with 10 mice from each group simultaneously, with no repetition on the experimental day.

### Statistical evaluation

Analyses were performed using the Statistical Package for the Social Sciences software (SPSS 22.0 Inc, Chicago, IL). Homogeneity of variance and normality distribution were verified using Levene and Shapiro-Wilk tests, respectively. Ordinary one-way ANOVA compared between-group differences of *in vivo* neutrophil migration, cytokines levels, synovitis score, nociception, and balance and motor coordination. Two-way ANOVA with repeated measures compared skin temperature and joint swelling with interaction between group (control, AIA, AIA + Cryotherapy) and initial and final periods of experimental analysis. Tukey’s HSD post-hoc test was performed when needed (α = 5% and 95% confidence interval). Data were expressed as mean ± SEM.

## Results

### *In vivo* neutrophil migration

**Fig 2:**
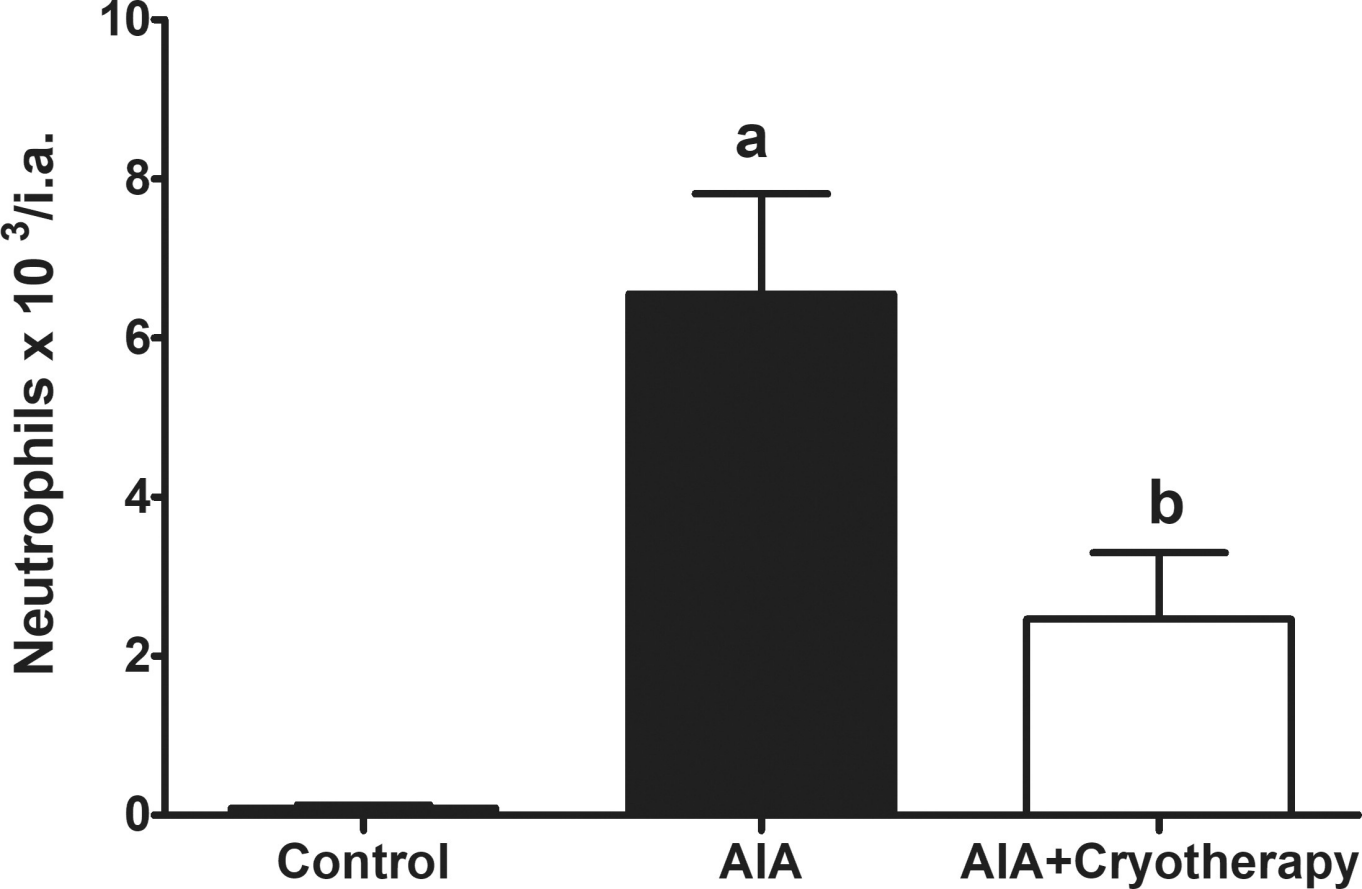
*In vivo* neutrophil migration in AIA model. Immunized C57BL/6 mice received i.a. injection of 100 μg of mBSA per joint and were treated with clinical-like cryotherapy (crushed ice bag). *In vivo* neutrophil migration was assessed in the final period of experimental analysis using a Neubauer chamber. Data expressed as mean ± SEM (n = 10). (a): Compared to control group; (b): Compared to AIA group.

Acute phase of AIA groups exhibited increased neutrophil recruitment into knee joint in the final period of experimental analysis compared with control group (mean difference: 6.46 neutrophils x 10^3^/i.a., Power = 0.99, F_2,27_: 14.00, P < 0.0001). AIA + Cryotherapy presented increased neutrophil recruitment into knee joint compared with control group (mean difference: 2.37 neutrophils x 10^3^/i.a., Power: 0.78; F_2,27_: 14.00, P > 0.05) and decreased compared with AIA group (mean difference: 4.09 neutrophils x 10^3^/i.a., Power: 0.75; F_2,27_: 14.00; P < 0.0001, Fig 2).

### Cytokines levels

**Fig 3:**
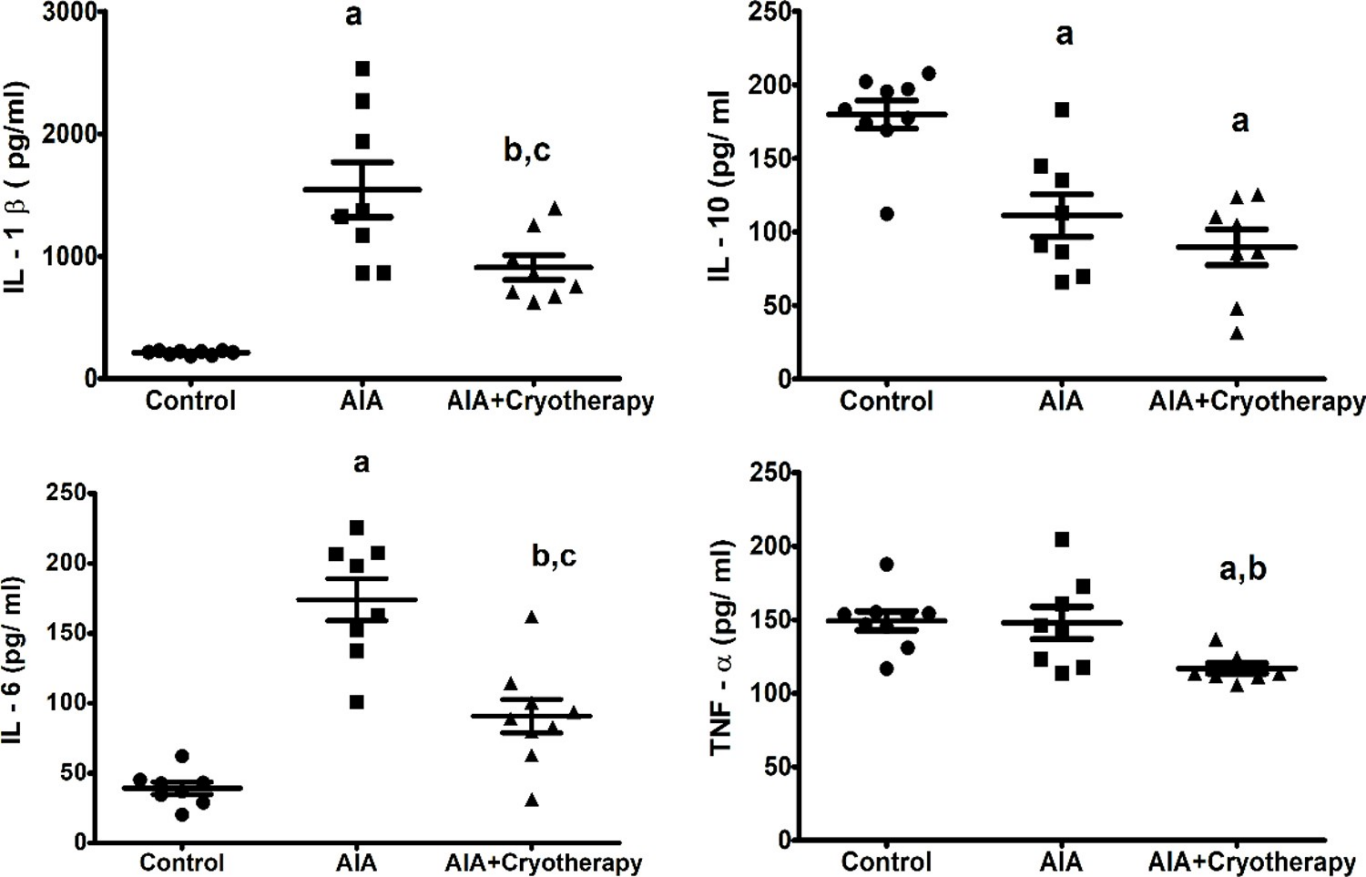
Levels of IL-1β, IL–10, IL-6, and TNF-α in AIA model. Immunized C57BL/6 mice received i.a. injection of 100 μg of mBSA per joint and were treated with clinical-like cryotherapy (crushed ice bag). Concentrations of (A) IL-1β, (B) IL-10, (C) IL-6, and (D) TNF-α were assessed in the final period of experimental analysis using ELISA Kit. Data expressed as mean ± SEM (n = 10). (a): Compared to control group; (b): Compared to AIA group; (c): Compared to AIA + Cryotherapy group.

In the final period of experimental analysis, concentrations of IL-1β (mean difference: 1331 pg/ml, Power = 0.99, F_2,22_: 24.64, P < 0.0001), IL-10 (mean difference: 68.68 pg/ml, Power = 0.99, F_2,22_: 15.91, P < 0.0001), and IL-6 (mean difference: 134.9 pg/ml, Power = 1.00, F_2,22_: 34.01, P < 0.0001) increased in AIA group compared with control group (Fig 3A–3C). In AIA + Cryotherapy group, IL-1β (mean difference: 635.9 pg/ml, Power = 0.79, F_2,22_: 24.64, P < 0.0001), IL-6 (mean difference: 83.34 pg/ml, Power = 0.98, F_2,22_: 34.01, P < 0.0001), and TNF-α (mean difference: 31.02 pg/ml, Power = 0.89, F_2,22_: 5.814, P = 0.0094) decreased compared with AIA group. IL-10 (mean difference: 90.25 pg/ml, Power = 0.99, F_2,22_: 15.91, P < 0.0001) and TNF-α (mean difference: 32.38 pg/ml, Power = 0.99, F_2,22_: 5.814, P = 0.0094) also decreased in the AIA + Cryotherapy group compared with control group (Fig 3B).

### Evaluation of joint inflammation

**Fig 4:**
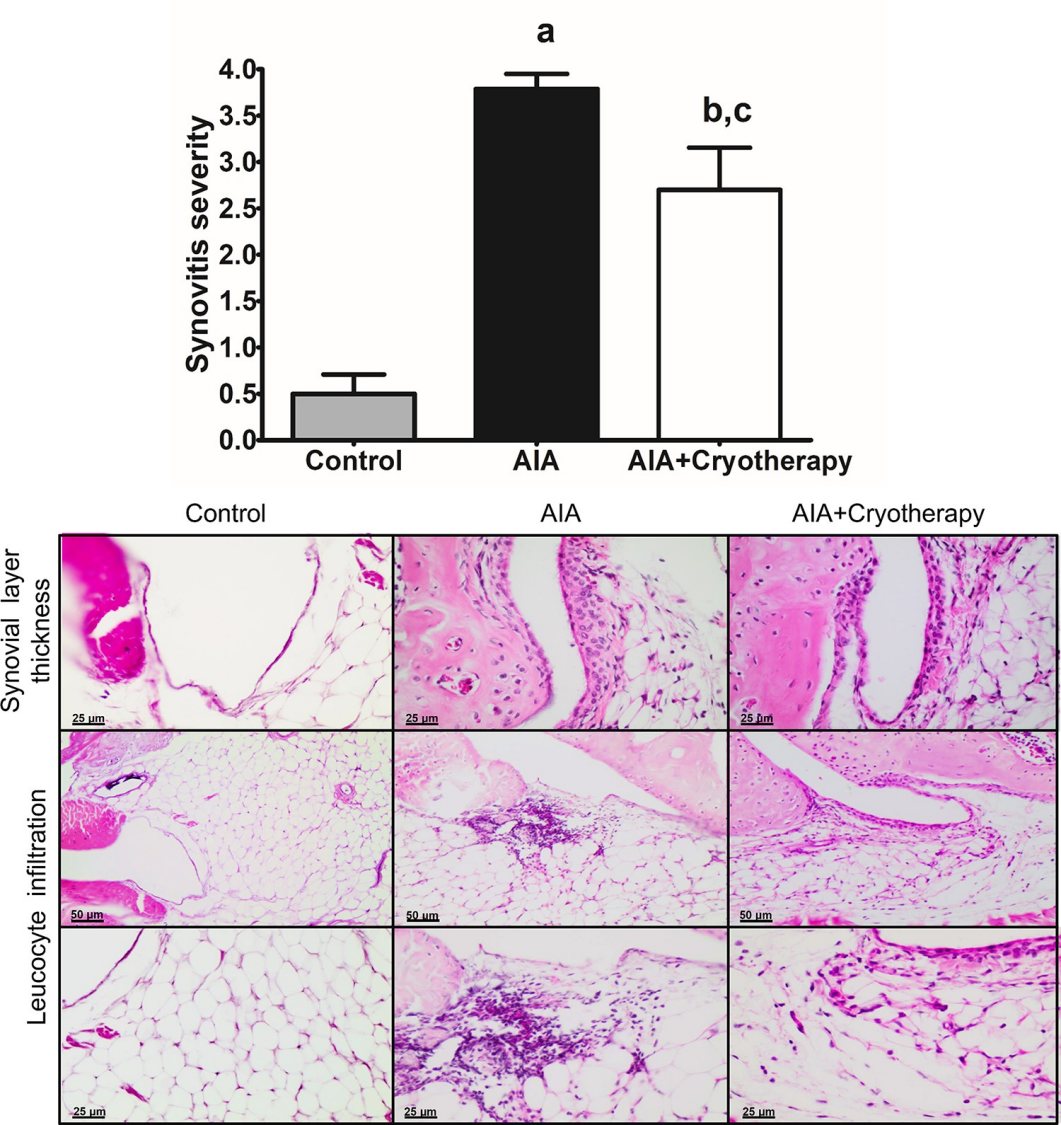
Synovitis scores in AIA model. **A:** Synovitis scores of acute joint pathology and synovial inflammation in the final period of experimental analysis. Data expressed as mean ± SEM (n = 10). (a): Compared to control group; (b): Compared to AIA group; (c): Compared to AIA + Cryotherapy group. **B:** Representative images of knee joint sections stained with H&E and respective histopathological scores.

Histological analysis of joint sections showed severe synovitis score in AIA group compared with control group (mean difference: 3.288, Power = 1.00, F_2,21_: 30.31, P < 0.0001, Fig 4A). Neutrophil infiltration in synovium and peri-articular tissues in the final period of experimental analysis showed a moderate synovitis score in AIA + Cryotherapy group compared with AIA (mean difference: 1.088, Power = 0.68, F_2,21_: 30.31, P < 0.0001) and control group (mean difference: 2.200, Power = 0.99, F_2,21_: 30.31, P < 0.0001, Fig 4A). Morphologically, scores indicated increased synovial layer thickness and cellular density caused by inflammatory leukocyte infiltration (Fig 4B).

### Dorsal flexion of tibiofemoral joint: Assessment using a modified electronic pressure-meter test for mice

**Fig 5:**
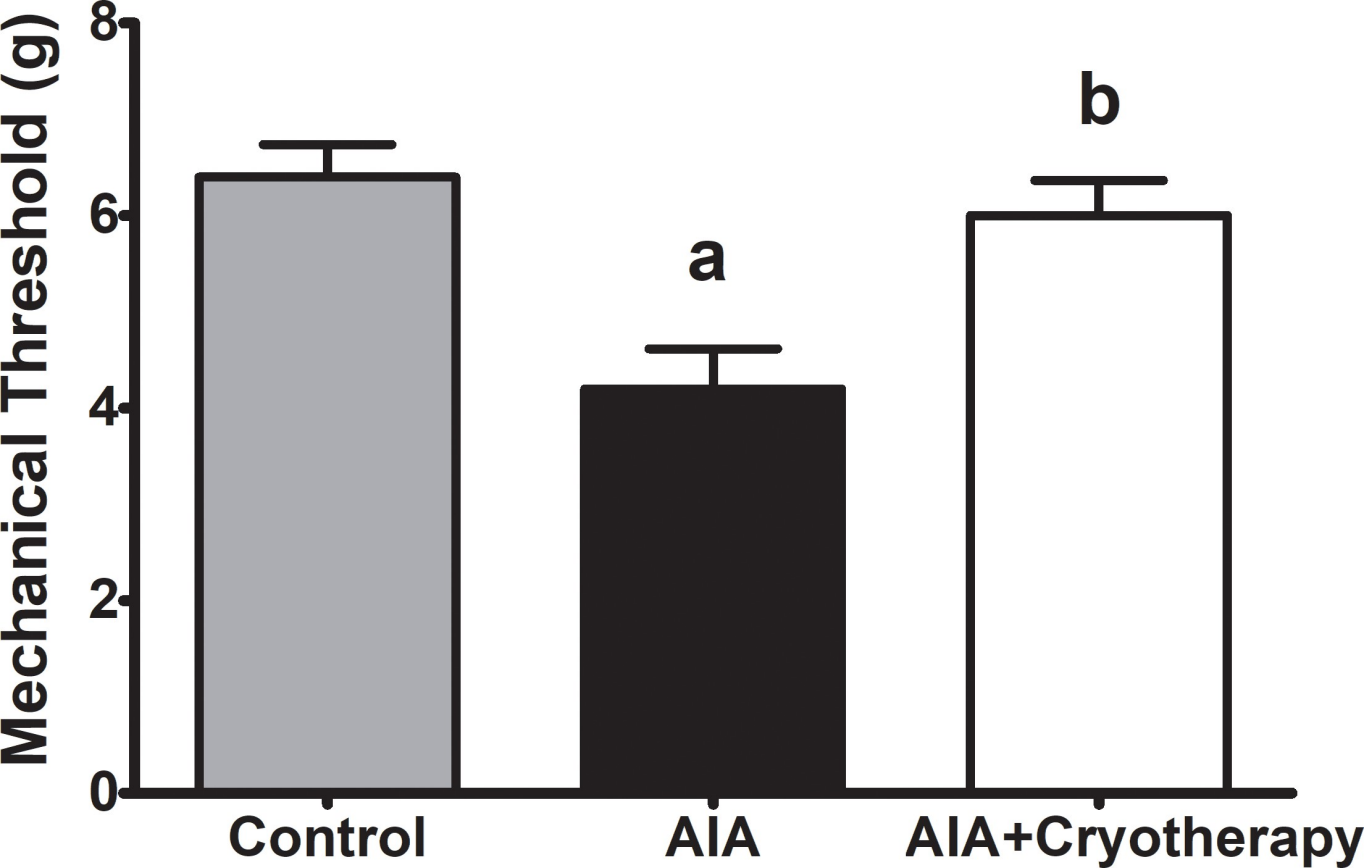
Mechanical threshold in AIA model. Immunized C57BL/6 mice received i.a. injection of 100 μg of mBSA per joint and were treated with clinical-like cryotherapy (crushed ice bag). Mechanical threshold was assessed using an electronic Von Frey esthesiometer in the final period of experimental analysis. Data expressed as mean ± SEM (n = 10). (a): Compared to control group; (b): Compared to AIA group.

Mechanical threshold reduced in AIA group compared with control group (mean difference: 2.200 g, Power = 0.96, F_2,27_: 9.758, P = 0.0006). On the other hand, mechanical threshold of AIA + Cryotherapy group increased compared with AIA group (mean difference: 1.800 g, Power = 0.87, F_2,27_: 9.758, P = 0.0006, Fig 5).

### Joint swelling (edema)

**Fig 6:**
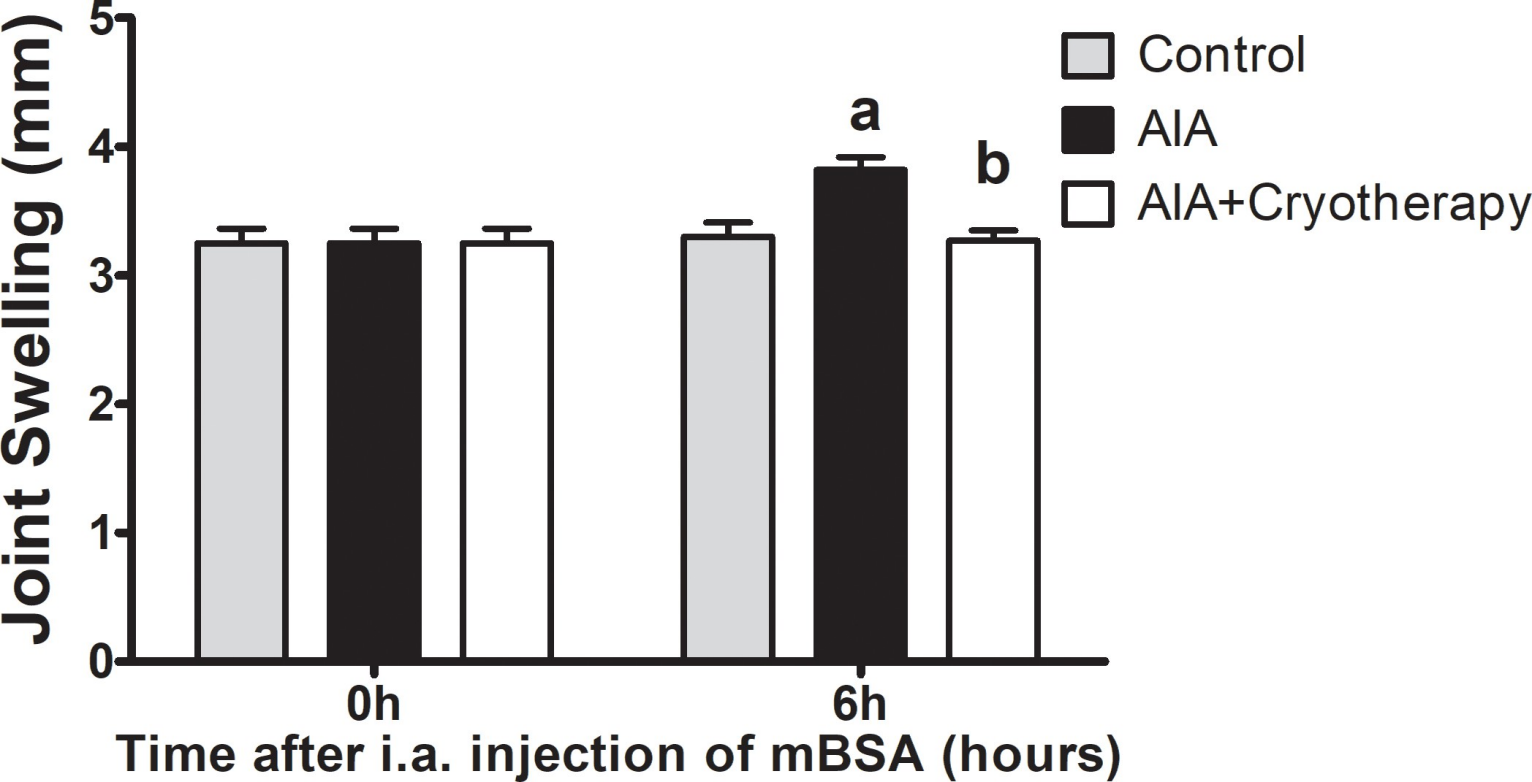
Joint swelling in AIA model. Immunized C57BL/6 mice received i.a. injection of 100 μg of mBSA per joint and were treated with clinical-like cryotherapy (crushed ice bag). Joint swelling (mm) was assessed using a digital caliper in the initial and final periods of experimental analysis. Data expressed as mean ± SEM (n = 10). (a): Compared to control group; (b): Compared to AIA group.

Knee joint swelling increased in AIA group compared with control group (mean difference: 0.52 mm, Power = 0.89, F_2,27_: 3.01, P < 0.01) in the final period of experimental analysis. Joint swelling was reduced in AIA + Cryotherapy group compared with AIA group (mean difference: 0.55 mm, Power = 0.95, F_2,27_: 3.01, P < 0.001), but no differences were observed compared with control group (mean difference: 0.03 mm, Power = 0.05, F_2,27_: 3.01 P > 0.05, Fig 6).

### Knee skin temperature

No difference in skin temperature of right knee joint was observed among groups (F_2,27_: 1.38, P > 0.05).

### Gait function—balance and motor coordination

**Fig 7:**
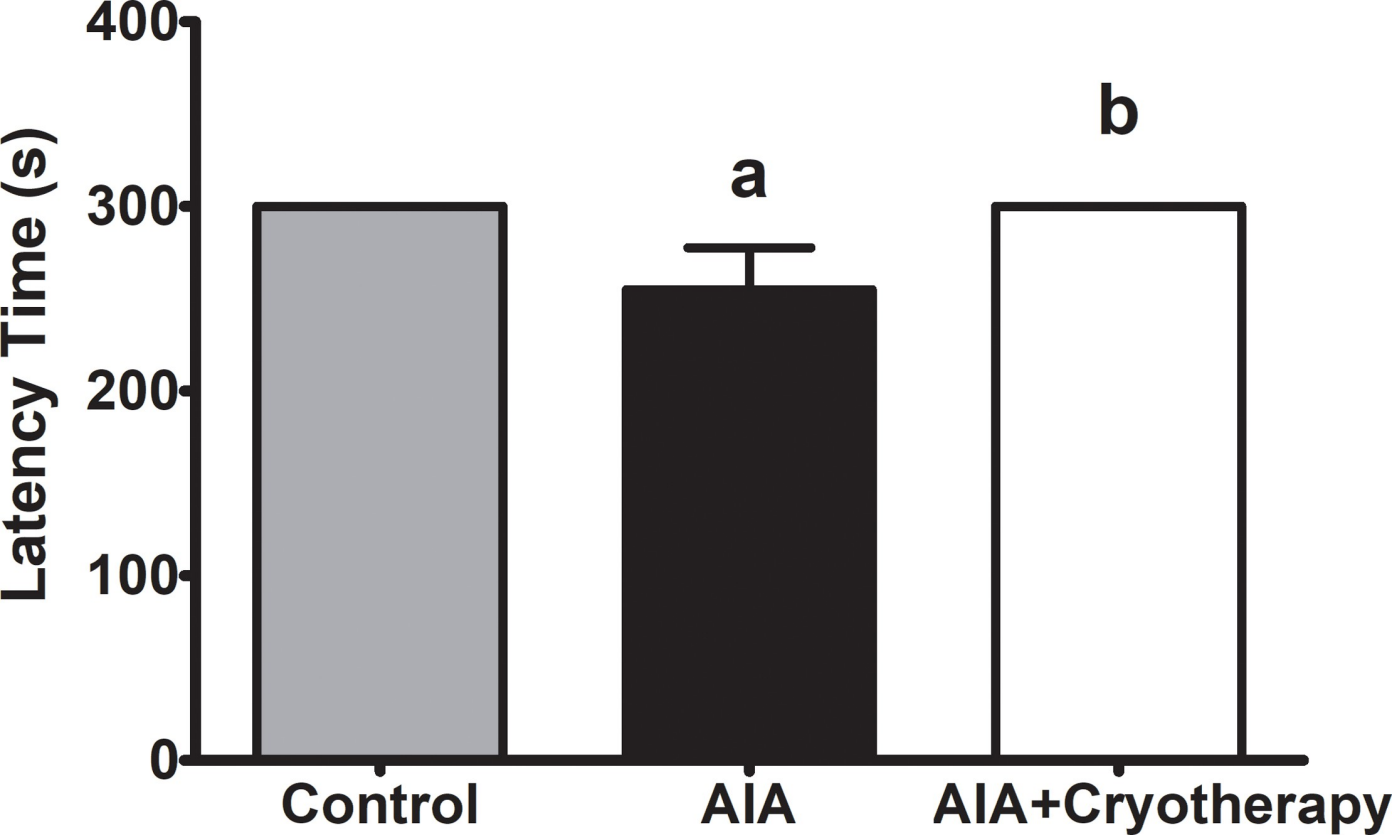
Gait function—balance and motor coordination in AIA model. Immunized C57BL/6 mice received i.a. injection with 100 μg of mBSA per joint and were treated with clinical-like cryotherapy (crushed ice bag). Balance and motor coordination were assessed using the rotarod test in the final period of experimental analysis. Data expressed as mean ± SEM (n = 10). (a): Compared to control group; (b): Compared to AIA group.

Latency time was shorter in AIA group than in control group (mean difference: 45.11 s, Power = 0.55, F_2,26_: 4.45, P = 0.02). In AIA + Cryotherapy group, latency time was higher than in AIA group (mean difference: 45.11 s, Power = 0.55, F_2,26_: 4.45, P = 0.02) and similar to control group (Mean difference: 0.00 s, Power = 0.05, F_2,26_: 4.45, P = 0.02, Fig 7).

## Discussion

Cryotherapy improved balance and motor coordination and reduced inflammatory signs (synovitis severity), pain, and joint swelling in mice with acute knee arthritis. Inflammatory signs (i.e., neutrophil migration, cytokines levels, and joint inflammation [synovitis severity]) are related to systemic inflammatory response of arthritis [38]. Moreover, the AIA experimental model is adequate for experimental studies and reproducible, with several histopathological characteristics similar to human rheumatoid arthritis [3,25].

Cryotherapy reduced neutrophil migration to synovial fluid. In humans, joints with arthritis present high levels of neutrophils, which may damage cartilage, bones, and tissue through secretion of proteases and metabolites. In addition, neutrophils stimulate inflammation by releasing cytokines, chemokines, leukotrienes, and prostaglandins [26–29]. The decrease in neutrophil migration was accompanied by pro-inflammatory cytokines IL-1β, IL-6, and TNF-α in synovial fluid, confirming the beneficial effect of clinical-like cryotherapy in controlling acute inflammation in knee arthritis. Cryotherapy may also downregulate important enzymatic pathways; for example, cytokines, cartilage-degrading enzymes, and proangiogenic factors involved in joint inflammation and destruction [7]. Cryotherapy did not decrease IL-10 levels in the present study, suggesting its action against progression of acute joint inflammation and demonstrating that IL-10 is beneficial as anti-inflammatory cytokine; however, the action pathway of IL-10 needs to be better investigated. Cytokines level (members of IL-1 family, IL-6 and its receptor, IL-17, IL-20, IL-21, and IL-23) increases in early stages of arthritis and decreases during regression of inflammation [30,31]. Histological analysis revealed that cryotherapy also reduced the inflammatory state by reducing leukocyte infiltration and reducing thickening of the synovial membrane.

Our results also showed the benefits of clinical-like cryotherapy in reducing pain and joint swelling. Pain resulting from AIA in mice depends on a cytokine cascade [18]. Joint swelling is common in several types of arthritis and is caused by synovial inflammation, increasing permeability of endothelial cells and plasma extravasation [32,33].

Inflammatory and nociceptive effects of arthritis may induce balance and motor abnormalities [34], despite no association between motor coordination and pain was found in our study. Therefore, we suggest that the effects of clinical-like cryotherapy contributed to preserving gait function. Cryotherapy has been used in clinical rehabilitation to reduce joint pain, swelling, degeneration, and inflammation in patients with arthritis induced by post-sports injury and several rheumatic joint diseases [35–38]. In addition, cryotherapy can be prescribed in isolation or associated with other therapies and may be well-accepted by patients with knee osteoarthritis [37]. According to Ogura et al., [37], the 20-min ice pack protocol simulates clinical treatment, and the effect of short-term cryotherapy benefits pain intensity.

Most studies investigating the effects of cryotherapy on acute arthritis used a protocol of longer duration or higher intensity than performed in clinical practice. Results of our cryotherapy protocol (based on clinical practice) indicate that new protocols should be developed, and other types of cryotherapy might be associated with treatment of rheumatoid arthritis. Several action mechanisms have been proposed to explain the effects of cryotherapy on inflammatory process of joints [7,8] and should be further studied. In the study by Ramos et al. [38], cryotherapy (three sessions of 30 min every two hours) decreased inflammatory processes (mRNA levels of TNF-α, NF-κB, TGF-β, MMP-9, and macrophage percentage) in the first 48 hours after injury. Also, Siqueira et al. [39] demonstrated that multiple applications of cryotherapy after muscle injury (30 min three times per day, on the day of the injury, and for two days after injury) reduced oxidative stress in rats. In another study [40], ice packs (30 min once per day for 10 days) reduced cell infiltrate and synovial hyperplasia in a rabbit model of zymosan-induced arthritis. Clinical-like cryotherapy also reduced joint synovial inflammation of anterior cruciate ligament transection-induced knee osteoarthritis [9]. Cryotherapy activates efferent parasympathetic neurons to release acetylcholine. These ligand-receptor interactions may inhibit NF-kB pathway and subsequently decrease pro-inflammatory cytokines, oxidative stress agents, and gene transcription of adhesion molecules [41–44]. Therefore, molecular, pro-inflammatory, and cytokine pathways should be studied to understand the anti-inflammatory properties in inflammatory rheumatic diseases.

Our study provides a new contribution on the benefits of clinical-like cryotherapy for treating acute knee arthritis in an animal model. Cryotherapy is also used empirically as complementary treatment for rheumatoid arthritis and present good tolerance compared with corticosteroids or non-steroidal anti-inflammatory drugs; however, protocols were not standardized yet [7].

## Conclusions

Two 20-min clinical-like cryotherapy sessions (the first session applied immediately after i.a. injection of mBSA and the second applied after 2 hours after the first session) decreased inflammatory signs (i.e., neutrophil migration and synovitis severity), pain, and joint swelling, and improved balance and motor coordination in mice in the acute phase of arthritis.

### Study limitations

This study is not free of limitations. No sham or placebo group was included in the experimental analysis. Second, pain assessment at baseline and knee temperature immediately after ice pack removal were not performed and could clarify the role of pain and skin cooling after cryotherapy. Third, we did not compare cryotherapy protocol with other protocols for arthritis. Therefore, future studies are needed to clarify these topics in animal models.

## Abbreviations

AIA: Antigen-induced arthritis
mBSA: Methylated bovine serum albumin
CFA: Freund´s complete adjuvant
PBS: Phosphate-buffered saline
EDTA: Ethylenediaminetetraacetic acid
ELISA: Enzyme-linked immunosorbent assay
H&E: Hematoxylin and eosin
